# Myeloid‐Derived CD38 Mediates Age‐Related Endometrial Aging Through NAD ^+^ Depletion

**DOI:** 10.1111/acel.70356

**Published:** 2025-12-28

**Authors:** Lun Hua, Luting Liu, Dengfeng Gao, Lulu Ma, Xianyang Jin, Liuyong Lu, Shangbo Tian, Xuemei Jiang, Chao Jin, Bin Feng, Lianqiang Che, Shengyu Xu, Yan Lin, Long Jin, Yong Zhuo, Mingzhou Li, De Wu

**Affiliations:** ^1^ Animal Nutrition Institute Sichuan Agricultural University Chengdu Sichuan China; ^2^ Animal Nutrition and Efficient Feed Utilization Key Laboratory of Sichuan Province Sichuan Agricultural University Chengdu Sichuan China; ^3^ Key Laboratory for Animal Disease‐Resistant Nutrition of the Ministry of Education of China Sichuan Agricultural University Chengdu Sichuan China; ^4^ State Key Laboratory of Swine and Poultry Breeding Industry, College of Animal Science and Technology Sichuan Agricultural University Chengdu Sichuan China

**Keywords:** CD38, endometrial aging, macrophage, NAD^+^

## Abstract

Against the backdrop of the global trend toward delayed childbearing, elucidating the mechanisms underlying uterine aging has emerged as a critical biomedical priority for addressing age‐related implantation failure. Through unbiased global metabolomic profiling of peri‐implantation uteri across different ages in mice, we identified nicotinamide adenine dinucleotide (NAD^+^) depletion as a hallmark metabolic feature of endometrial aging. Single‐cell RNA sequencing further revealed an expansion of senescent stromal cell populations, which was accompanied by a decline in NAD^+^ levels. Supplementation with NAD^+^ precursors alleviated age‐related stromal senescence and endometrial dysfunction, thereby restoring the uterus' implantation competence. Mechanically, we demonstrate that CD38 derived from myeloid serves as a principal driver of uterine NAD^+^ depletion; this process accelerates stromal senescence and impairs uterine receptivity. These findings establish CD38 as a central physiological integrator that links NAD^+^ metabolism to uterine function and highlight it as a promising target for rejuvenation strategies aimed at improving reproductive outcomes in women of advanced maternal age.

## Graphical abstract

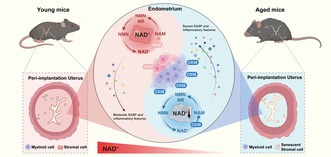

## 1 Introduction

The global prevalence of delayed childbearing is increasing, with the average age at first childbirth in developed countries rising by approximately 1 year per decade since 1970 (Fauser et al. 2024). Although assisted reproductive technologies can partially offset age‐related declines in oocyte and embryo quality, pregnancy ratios among women over 40 years old remain persistently low (Khalil et al. 2013; Shapiro et al. 2016; Magnus et al. 2019; Frick 2021). The human endometrium, serving as a transient implantation niche, supports placental development through dynamic nutritional and immunological interactions (Gellersen and Brosens 2014; Shibata et al. 2024). Aging is associated with endometrial dysfunction, characterized by abnormal endometrial thickness, chronic inflammation, and attenuated hormonal responsiveness (Woods et al. 2017; Secomandi et al. 2022). However, the precise cellular and metabolic alterations that drive these age‐related changes, along with their implications for embryo implantation, remain incompletely understood. Given the increasing trend in maternal age at first birth, elucidating the mechanisms underlying endometrial aging has become a critical biomedical priority to identify novel therapeutic strategies.

In mammals, the majority of pregnancy losses occur during the peri‐implantation period (Busnelli et al. 2021). Successful early pregnancy necessitates the coordinated progression of embryo development, uterine receptivity (encompassing decidualization in humans), and implantation (Cha et al. 2012; Gellersen and Brosens 2014). The receptive phase, known as the window of implantation (WOI), is of short duration. For instance, in mice, uterine receptivity is restricted to a few hours on Day 4 of pregnancy (with Day 1 defined as the detection of a vaginal plug) (Wang and Dey 2006). During this window, stromal, epithelial, and immune cells within the endometrium coordinate to establish a permissive microenvironment, and precise embryo‐endometrium crosstalk is essential for successful implantation and subsequent pregnancy maintenance (Jin et al. 2021; Yang et al. 2023; Xu et al. 2025). Although extensive work has been conducted to characterize the transcriptomic alterations associated with endometrial aging during the WOI (Tanikawa et al. 2017; Kawamura et al. 2021; Wang et al. 2025), comprehensive insights into cell type‐specific changes occurring in this timeframe have long been lacking.

The endometrium undergoes cyclic shedding, regeneration, and differentiation throughout the reproductive lifespan under the regulation of estrogen and progesterone (Bergqvist and Fernö 1993; Garcia‐Alonso et al. 2021). This high‐turnover state imposes substantial energetic demands on the tissue, rendering it particularly susceptible to accelerated aging. Consequently, the endometrium serves as an attractive model for dissecting the spatiotemporal dynamics of cellular senescence (Wang et al. 2025). Metabolic adaptations during decidualization and implantation are critical for supporting conceptus development (King 2000; Yu et al. 2024); therefore, systematic profiling of age‐related metabolomic changes and intercellular metabolic coupling during the WOI is urgently required.

Nicotinamide adenine dinucleotide (NAD^+^), a key cofactor in energy metabolism and cellular signaling pathways, is closely associated with cellular senescence (Covarrubias et al. 2021; Chini et al. 2024). Age‐related declines in NAD^+^ levels have been documented in multiple tissues in both rodents and humans (Houtkooper et al. 2010; Song et al. 2025). Restoration of NAD^+^ levels through precursor supplementation can attenuate functional deterioration in a mouse model (Bertoldo et al. 2020; Miao et al. 2020). Despite evidence of the accumulation of senescent cells in the aged endometrium and the established critical role of NAD^+^ metabolism in implantation (Li et al. 2016, 2022; Loid et al. 2024), the question of whether and how NAD^+^ homeostasis regulates endometrial receptivity remains unresolved.

In this study, we identify NAD^+^ depletion as a defining metabolic feature of endometrial aging. Notably, supplementation with NAD^+^ precursors not only ameliorates age‐related endometrial dysfunction but also restores implantation competence. We further demonstrate that CD38 (a key NAD^+^‐consuming enzyme) is enriched in macrophages and thereby orchestrates uterine NAD^+^ loss while impairing stromal proliferative capacity. These findings collectively reveal a macrophage‐mediated metabolic axis underlying reproductive aging and nominate CD38 as a potential therapeutic target for improving pregnancy outcomes in women of advanced maternal age.

## 2 Results

### 2.1 Altered NAD ^+^ Metabolism in the Aging Endometrium

**FIGURE 1:**
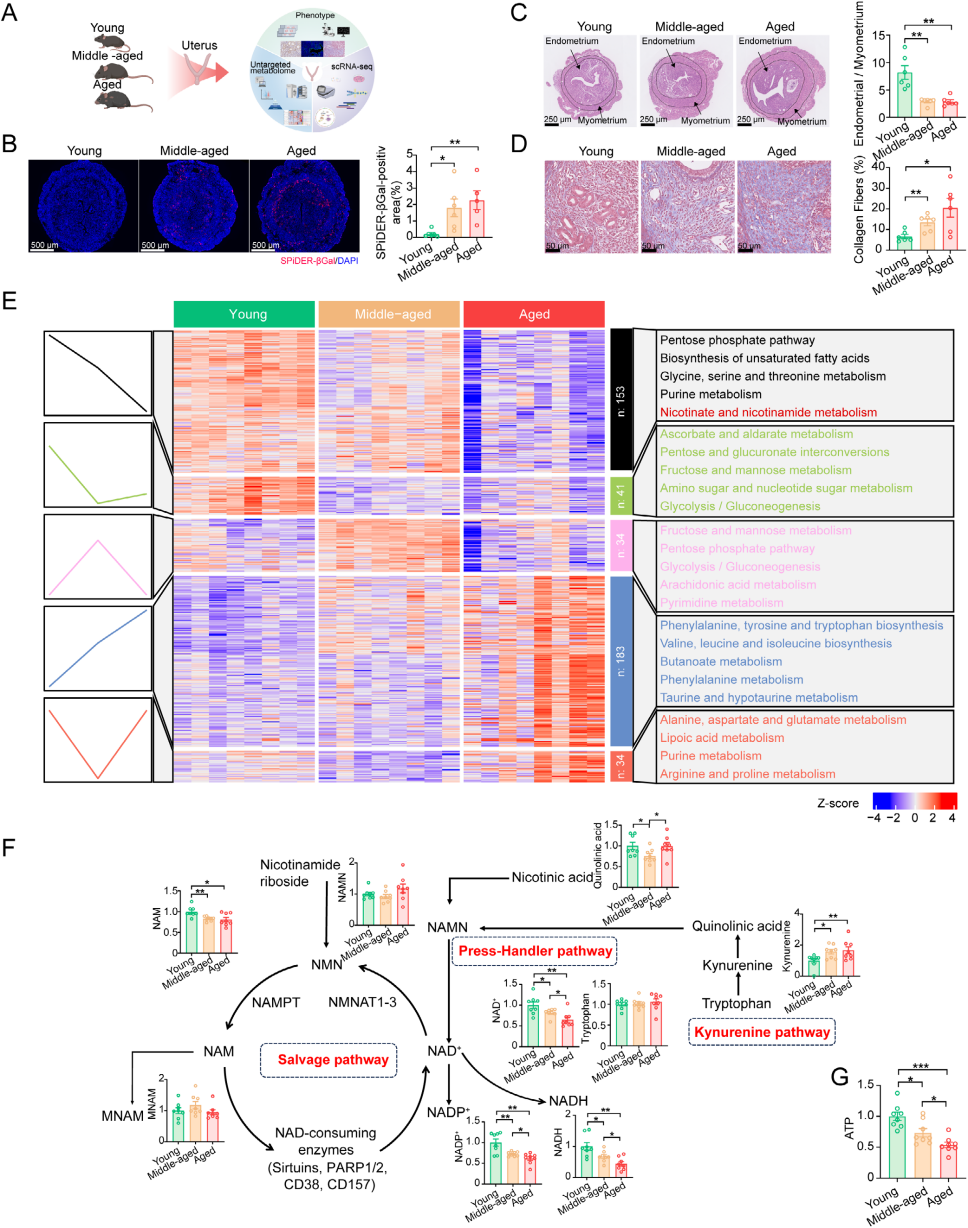
Aging endometrium exhibits altered NAD^+^ metabolism. (A) Schematic diagram illustrating the experimental setup. “Young” denotes 3‐month‐old; “Middle‐aged” denotes 8‐month‐old; “Aged” denotes 12‐month‐old. Sample were collected at the peri‐implantation stage (embryonic day 4, E4). Created with BioRender.com. (B) Immunofluorescence staining of SPiDER‐βGal in the endometrium from 3‐, 8‐, and 12‐month‐old mice. Representative images are shown on the left; quantitative data for the percentage of SPiDER‐βGal‐positive areas are shown on the right (*n* = 6). (C) H&E staining of uterine tissues from 3‐, 8‐, and 12‐month‐old mice. Representative images are shown on the left; quantitative data for the endometrium‐to‐myometrium ratio are shown on the right (*n* = 6). (D) Masson's trichrome staining of uterine tissues from 3‐, 8‐, and 12‐month‐old mice. Representative images are shown on the left; quantitative data for the fibrotic positive areas are shown on the right (*n* = 6). (E) Heatmap showing differentially abundant metabolites in uterine tissues from 3‐, 8‐, and 12‐month‐old mice at the peri‐implantation stage (*n* = 8). (F) Levels of ATP in uterine tissues from 3‐, 8‐, and 12‐month‐old mice (*n* = 8). Data are presented as mean ± SEM. Unless otherwise specified, comparisons were conducted using one‐way analysis of variance (ANOVA) followed by Tukey's post hoc test. Statistical significance was set at *p* < 0.05, and the significance of difference is expressed as: *p* < 0.05 (*), *p* < 0.01 (**), and *p* < 0.001 (***). Each symbol represents one biological replicate.

To characterize the phenotypic and molecular features associated with endometrial aging, we collected uterine tissues from young (3 months), middle‐aged (8 months), and aged (12 months) mice at the peri‐implantation stage (embryonic day 4, E4) (Figure 1A). The ages of these mice approximately correspond to 20, 35, and 41 human years, respectively. Consistent with established aging phenotypes (Loid et al. 2024; Wang et al. 2025), we observed a marked accumulation of senescent cells in the aged endometria (Figure 1B). We also observed that aging is associated with a decrease in endometrial thickness and an increase in fibrotic deposition (Figure 1C,D), both of which reflect the deterioration of the endometrial architecture essential for implantation.

Using an untargeted metabolomics approach, we identified a total of 843 metabolites in uterine tissues across three different age groups, and these metabolites could be categorized into five distinct clusters. Tissue dysfunction is frequently associated with the reprogramming of amino acid and carbohydrate metabolism, encompassing glycolysis, the tricarboxylic acid (TCA) cycle, and the pentose phosphate pathway (Asadi Shahmirzadi et al. 2020; Chaleckis et al. 2016; Sebastiani et al. 2024). Consistent with observations in other tissues, our results demonstrate that amino acid and carbohydrate metabolism in the uterus undergoes profound age‐related alterations (Figure 1E). Pathway enrichment analysis revealed a pronounced age‐related decline in nicotinate and nicotinamide (NAM) metabolism (Figure 1F). Subsequently, we integrated these differential changes with NAD^+^‐targeted metabolic data for combined pathway analysis. Comparison of uteri from aged mice with those from young mice showed a substantial reduction in the levels of three key metabolites involved in nicotinate and NAM metabolism (i.e., NAD^+^, NADH, and NADP^+^) (Figure 1F). Given that NAD^+^ homeostasis is coupled to mitochondrial electron transport and ATP generation (Cantó et al. 2015; Xiao et al. 2018), we measured ATP levels and observed a progressive depletion with increasing age (Figure 1G). These findings suggest that NAD^+^ loss is a key metabolic feature of endometrial aging, which may potentially contribute to impaired endometrial receptivity.

### 2.2 Alterations in Cell Populations of the Aging Endometrium

**FIGURE 2:**
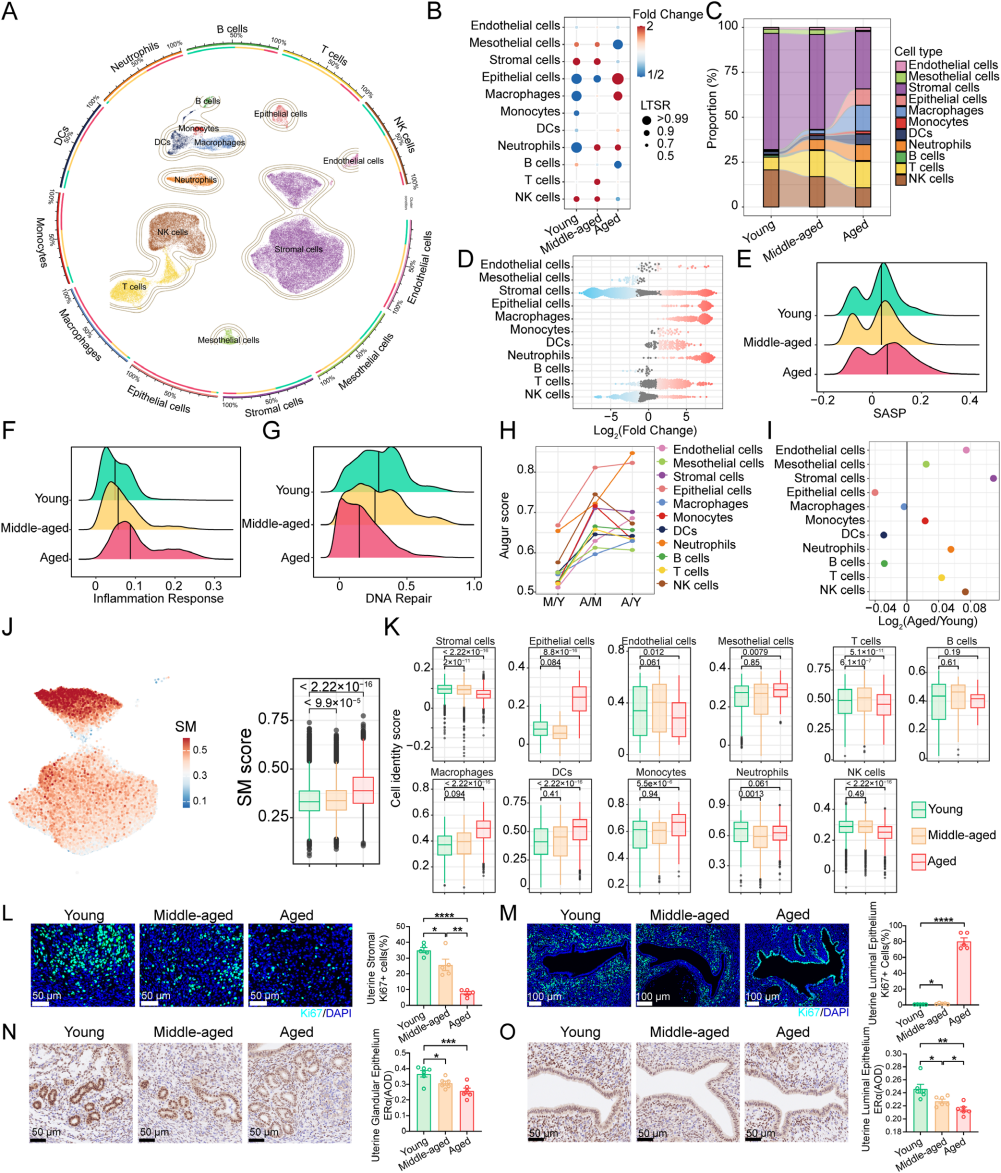
Cell type‐specific alterations in aged endometrium. (A) UMAP of 63,083 cells isolated from the peri‐implantation uteri of 3‐, 8‐, and 12‐month‐old mice. Major cell types were annotated based on marker gene expression. (B) Fold changes in cell‐type abundance with age, estimated using a Poisson generalized linear mixed model. (C) Sankey diagram showing changes in cellular composition across ages. (D) Bee‐swarm plot of log_2_ fold changes in cell‐type abundance between 3‐ and 12‐month‐old mice derived from Milo analysis; neighborhoods with spatial FDR ≤ 10% are colored. (E–G) Ridge plots comparing SASP (E), inflammatory response (F), and DNA repair (G) signature scores across the uteri of 3‐, 8‐, and 12‐month‐old mice. (H) Cell‐type aging responsiveness measured by Augur in the uteri of 3‐, 8‐, and 12‐month‐old mice (higher score indicates greater age‐related transcriptional perturbation). (I) Dot plot showing the log_2_ ratio of transcriptional noise (12‐ vs. 3‐month). (J) Senescence atlas: UMAPs colored by GSVA scores (GASM SenMayo) in stromal cells (left), boxplot of SM scores across age groups (right). Box plot showing the cell identity score of each cell type in the uteri of 3‐, 8‐, and 12‐month‐old mice. (L, M) Immunofluorescence staining of Ki67 in stromal cells (L) and luminal epithelial cells (L) from 3‐, 8‐, and 12‐month‐old mice. Representative images are shown on the left; quantitative data for the percentage of Ki67‐positive cells are shown on the right (*n* = 5). (N, O) IHC staining of ERα in glandular (N) and luminal (O) epithelial cells of endometrium from 3‐, 8‐, and 12‐month‐old mice. Representative images are shown on the left; quantitative data for average optical density (AOD) are shown on the right (*n* = 6). Data are presented as mean ± SEM. Unless otherwise specified, comparisons were conducted using one‐way analysis of variance (ANOVA) followed by Tukey's post hoc test. The statistical significance is set to *p* < 0.05, and the difference significance is expressed as: *p* < 0.05 (*), *p* < 0.01 (**), and *p* < 0.0001 (****). Each symbol represents one biological replicate.

To comprehensively characterize the age‐related changes in cell populations resident in the uterus, we performed a single‐cell RNA sequencing (scRNA‐seq) survey of 63,083 high‐quality cells from the uteruses of young, middle‐aged, and aged mice. The uniform manifold approximation and projection (UMAP) visualization of these cells revealed 11 major cell types, which were annotated based on the expression of well‐recognized marker genes (Figure 2A, Figure S1A,B). Stromal cells (33,058 of 63,083; or 52.4%) and NK/T cells (18,232 of 63,083; or 28.9%) constituted the most abundant cell populations. A generalized linear mixed model (Yoshida et al. 2022) revealed age‐related increases in epithelial cells, macrophages, and neutrophils, and age‐related decreases in stromal and NK cells (Figure 2B,C). The Milo algorithm (Dann et al. 2022) depicted similar results (Figure 2D, Figure S2A,B).

Consistent with the accumulation of senescent cells in the aged endometrium (Loid et al. 2024; Wang et al. 2025), scRNA‐seq analysis revealed age‐related upregulation of senescence‐associated secretory phenotype (SASP) associated genes and inflammatory mediators (Figure 2E,F), as well as downregulation of DNA repair markers in the uterus (Figure 2G). Augur‐based cell perturbation analysis identified epithelial cells, stromal cells, and neutrophils as highly age‐responsive populations (Figure 2H). Measurement of transcriptional noise, a well‐recognized hallmark of aging (Androvic et al. 2020; Bartz et al. 2023), revealed that stromal cells exhibited the most substantial age‐related increase in cell‐to‐cell transcriptional variability (Figure 2I, Figure S2C,D). To further construct a senescence atlas, we computed a unified senescence score (USS) by integrating six reference gene sets (i.e., SenMayo, CellAge, GenAge, Senescence Eigengene approach, SASP, and inflammation response) using the ssGSVA approach (see Methods) (Li et al. 2025). Stromal cells in aged uteri also exhibited markedly elevated USS compared with those in young counterparts (Figure 2J).

Loss of cell identity is a common feature of cellular aging (Hu et al. 2024; Yang, Chen, et al. 2024; Yang, Liu, et al. 2024), consistent with our results showing that compromised cell identity was evident in stromal cells (Figure 2K). Interestingly, we found that, in contrast to the decline observed in stromal cells, epithelial cells exhibited higher cell identity scores with aging (Figure 2K). This inverse pattern between stromal and epithelial cell identity was further supported by increased protein abundance of Ki67 (a marker of proliferation) (Figure 2L,M), indicating that the expansion of senescent stromal populations alongside hyperproliferative epithelial cells represents a characteristic feature of aged uteri. Steroid hormones drive epithelial cells from the proliferation phase to the differentiation phase during WOI (Bergqvist and Fernö 1993; Garcia‐Alonso et al. 2021). Our observations revealed that, in comparison with young mice, the expression of estrogen receptor α (ERα) was significantly lower in the endometrial epithelium of aged mice (Figure 2N,O). These findings suggest that age‐related impairments in hormonal responsiveness contribute to the hyperproliferation of endometrial epithelial cells, which may potentially compromise endometrial receptivity.

### 2.3 Age‐Related Decline of Stromal NAD ^*+*^ Impairs Uterine Decidualization

**FIGURE 3:**
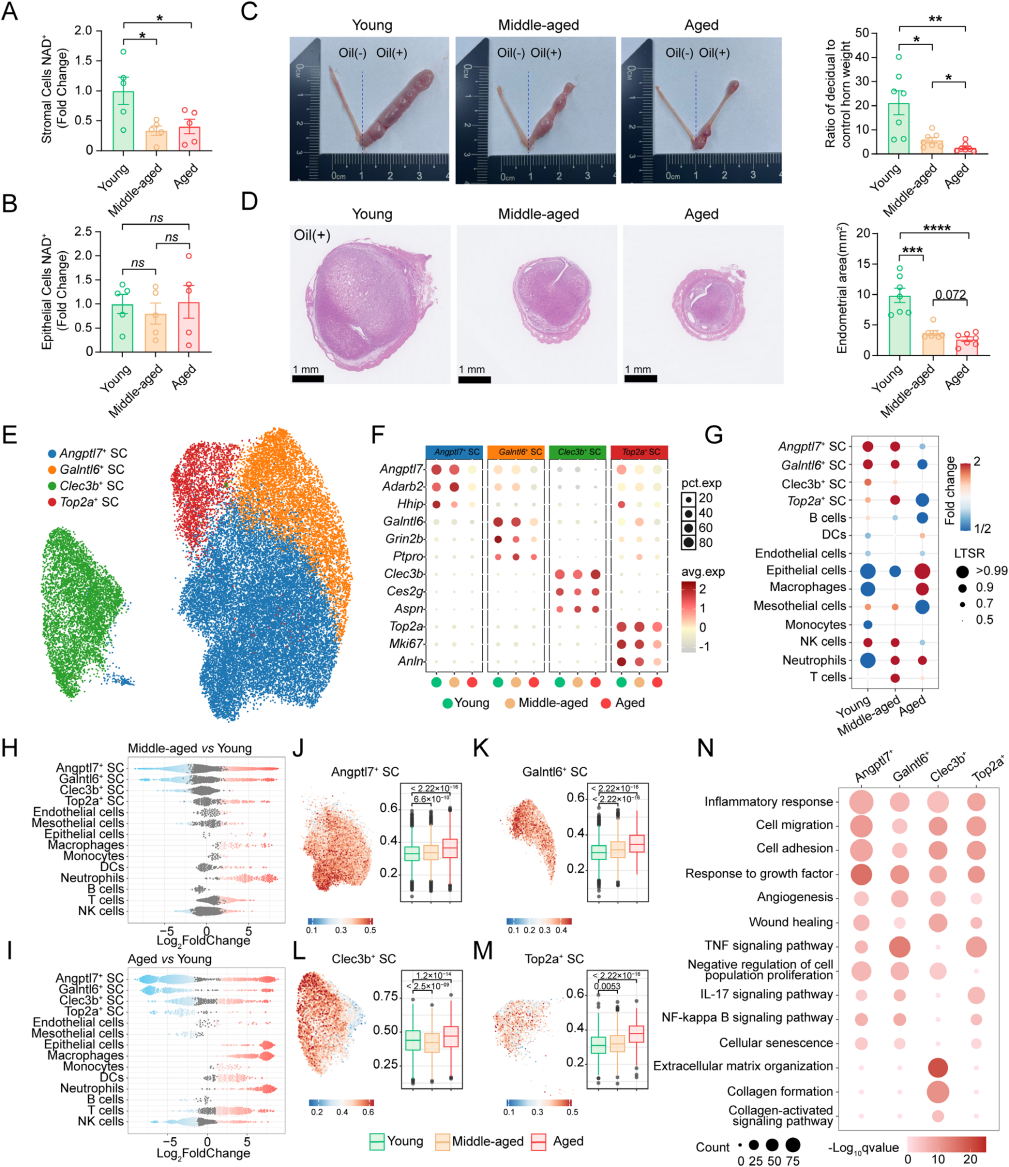
Stromal cell heterogeneity, aberrant epithelial proliferation, and age‐related decline in stromal NAD^+^ levels. (A, B) Levels of NAD^+^ in the stromal cells (M) and epithelial cells (N) from 3‐, 8‐, and 12‐month‐old mice at the peri‐implantation stage (*n* = 5). (C) Artificial decidua formation in the uteri of 3‐, 8‐, and 12‐month‐old mice. Representative images are shown on the left and quantitative data for the ratio of the weight of the decidual uterine horn between different groups are shown on the right (*n* = 7). (D) H&E staining of uterine decidua. Representative images are shown on the left and quantitative data of the endometrium area are shown on the right (*n* = 7). (E) UMAP visualization of the subpopulations of stromal cells (SC). (F) Dot plot showing the expression of top marker genes for each stromal subcluster in the scRNA‐seq dataset from peri‐implantation uteri of 3‐, 8‐, and 12‐month‐old mice. (G) Fold changes in subpopulation abundance with age, estimated by a Poisson generalized linear mixed model. (H, I) Bee‐swarm plot of log_2_ fold changes in cell‐type abundance from Milo analysis; neighborhoods with spatial FDR ≤ 10% are colored. (J–M) Senescence atlas in stromal cells. UMAPs are colored by GSVA scores (GA); bar plots show the ratio of senescent (Sn) to non‐senescent (nSn) cells per subcluster (left), boxplot of SM scores across age groups (right). (N) Dot plot of representative upregulated Sn differentially expressed genes (DEGs) GO terms. Data are presented as mean ± SEM. Unless otherwise specified, comparisons were conducted using one‐way analysis of variance (ANOVA) followed by Tukey's post hoc test. Statistical significance was set at *p* < 0.05, and the significance of difference is expressed as: *p* < 0.05 (*), *p* < 0.01 (**), *p* < 0.001 (***), and *p* < 0.0001 (****). ns means no significant difference. Each symbol represents one biological replicate.

Endometrial stromal cells and epithelial cells are crucial for endometrial receptivity and undergo substantial metabolic changes during the WOI (Deryabin and Borodkina 2022; Yu et al. 2024). To characterize the regionalized metabolic alterations in NAD^+^ metabolism associated with endometrial aging, we isolated stromal cells and epithelial cells from uteri at the peri‐implantation stage. Notably, in comparison to epithelial cells, stromal cells derived from aged mice displayed a more marked reduction in NAD^+^ levels, thereby rendering them more vulnerable to the aging process (Figure 3A,B). Given the critical role of stromal cells in decidualization (Deryabin et al. 2020), we assessed functional responsiveness using an artificial decidualization model. Young mice exhibited a robust decidual response, as evidenced by a significant increase in the wet weight and size of the decidua in the stimulated right uterine horn compared to aged mice (Figure 3C,D). These results indicate that aging impairs the NAD^+^ metabolism and the proliferative capacity of stromal cells in the endometrium.

To further characterize the age‐related changes in subclass‐level variations, we re‐clustered stromal cells (SC) into four subtypes (Figure 3E,F). We observed that *Angptl7* ^+^ SC, *Galntl6* ^+^ SC, and *Top2a* ^+^ SC exhibited a gradual decrease with the progression of age (Figure 3G–I). To define molecular differences among these subclusters, we computed the USS to identify senescent cells. Compared with young mice, aged mice showed increased proportions of senescent cells in multiple stromal subclusters: *Angptl7* ^+^ SC (from 3.4% to 11.6%), *Galntl6* ^+^ SC (from 0.7% to 4.5%), *Clec3b* ^+^ SC (from 2.1% to 3.6%), and *Top2a* ^+^ SC (from 0.25% to 1.1%) (Figure 3J–M). Those subclusters exhibited markedly elevated USS compared with those in young counterparts (Figure 3J–M), and the differential genes of the four stromal cell clusters also exhibit characteristics of senescence (Figure 3N). Interestingly, *Clec3b* ^+^ SC showed significantly higher enrichment in terms including “collagen formation,” “extracellular matrix organization,” and “collagen‐activated signaling pathway” (Figure 3N). This is consistent with our histological observation of increased fibrotic deposition in the aged endometrium (Figure 1D), indicating that the changes of *Clec3b* ^+^ SC may be linked to fibrotic remodeling in the uteri of aged mice.

Collectively, these results suggest that the decline in NAD^+^ levels in stromal cells may be linked to impaired proliferative capacity of the endometrium, which contributes to impaired endometrial receptivity.

### 2.4 Restoring Uterine NAD ^+^ Homeostasis Alleviates Age‐Related Impairment of Endometrial Receptivity

**FIGURE 4:**
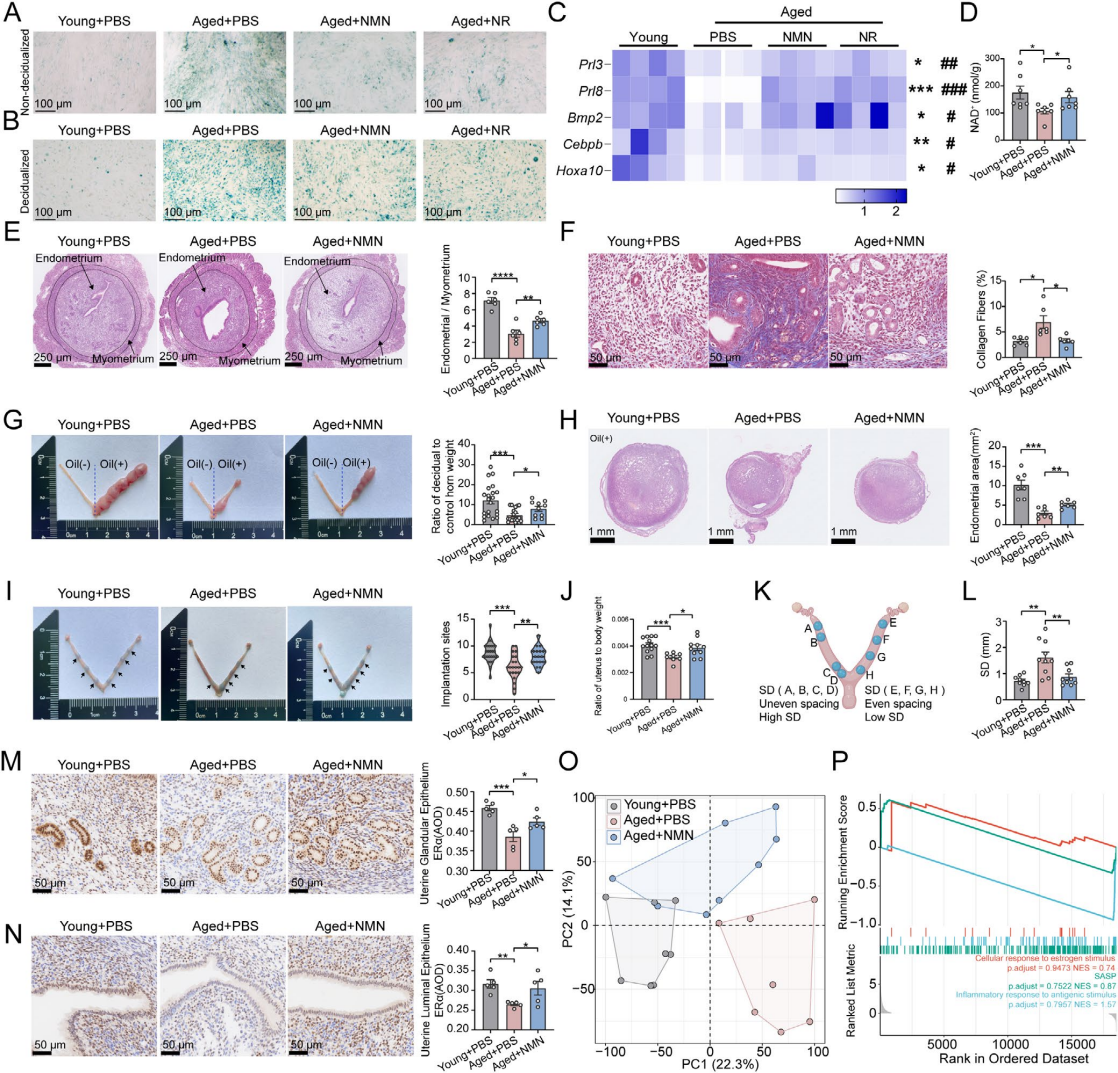
Restoring Uterine NAD^+^ homeostasis alleviates age‐related impairment of endometrial receptivity. (A, B) Representative SA‐β‐GAL staining images of primary stromal cells isolated from the uteri of young and aged mice, treated with NMN and NR during non‐decidualization (A) or decidualization (B). (C) Relative mRNA levels of decidualization markers in endometrial stromal cells treated with NMN and NR. (D) NAD^+^ levels in uterine tissues from aged mice that received daily injections of NMN (200 mg/kg body weight) for 2 weeks and were collected at the peri‐implantation stage (embryonic day 5, E5) (*n* = 7). (E) H&E staining of uteri from young, aged, and aged mice treated with NMN. Representative images are shown on the left and quantitative data for the ratio of endometrium and myometrium are shown on the right (*n* = 6). (F) Masson's trichrome staining of uterine tissues from young, aged, and aged mice treated with NMN. Representative images are shown on the left and quantitative data for fibrosis‐positive areas are shown on the right (*n* = 6). (G) Artificial decidua formation in the uteri of young, aged, and aged mice treated with NMN. Representative images are shown on the left, and quantitative data for the ratio of the weight of the decidual uterine horn between different groups are shown on the right (Young+PBS, *n* = 21; Aged+PBS, *n* = 21; Aged+NMN, *n* = 10). (H) H&E staining of uterine decidua. Representative images are shown on the left and quantitative data of the endometrium area are shown on the right (*n* = 7). (I) Implantation sites after injection of 2% Chicago blue dye on embryonic day 6 (E6). Representative images are shown on the left and quantitative data for the number of implantation sites are shown on the right (Young+PBS, *n* = 27; Aged+PBS, *n* = 22; Aged+NMN, *n* = 17). (J) Ratio of uterine weight to body weight on E5 (Young+PBS, *n* = 13; Aged+PBS, *n* = 8; Aged+NMN, *n* = 10). (K, L) Schematic illustration of the quantitation method for blastocysts spacing within uterine horns (K). Quantification of blastocyst spacing in uteri of mice on E6 (L) (*n* = 9–10). (M, N) IHC staining of ERα in glandular (M) and luminal (N) epithelial cells of the endometrium from young and aged and NMN‐treated aged mice. Representative images are shown on the left and quantitative data for the average optical density (AOD) are shown on the right (*n* = 5). (O) PCA of RNA‐seq data from uterine tissues from young, aged, and NMN‐treated aged mice. (P) GSEA analysis of the ERα‐related pathway, SASP, and inflammatory response between aged and NMN‐treated aged mice. Data are presented as mean ± SEM. Unless otherwise specified, comparisons among three or more groups were analyzed using one‐way analysis of variance (ANOVA) followed by Tukey's post hoc test. Statistical significance was set at *p* < 0.05, and the significance of difference is expressed as: *p* < 0.05 (*), *p* < 0.01 (**), *p* < 0.001 (***), and *p* < 0.0001 (****). Each symbol represents one biological replicate.

Given our observation of a marked impairment in NAD^+^ metabolism within the stromal cells of aged mice, we sought to investigate whether supplementation with NAD^+^ precursors could protect stromal cells against age‐related senescence. We isolated stromal cells from aged mice and subjected them to in vitro treatment with nicotinamide mononucleotide (NMN) or nicotinamide riboside (NR), respectively. Our findings demonstrated that both NAD^+^ precursor supplementation strategies effectively attenuated cellular senescence in both the non‐decidualized and decidualized states (Figure 4A,B) and upregulated the expression of five decidualization markers (Figure 4C).

To evaluate whether NAD^+^ replenishment restores uterine function in vivo, aged female mice were administered NMN for 2 weeks. NMN treatment normalized uterine NAD^+^ levels to a range comparable to that of young mice (Figure 4D). Notably, NMN reversed age‐related endometrial thinning and attenuated progressive fibrotic deposition (Figure 4E,F). Moreover, through an artificially induced decidualization model, we confirmed that NMN enhances the proliferative capacity of the endometrium (Figure 4G,H). Collectively, these findings indicate that NMN supplementation ameliorates the features of endometrial aging.

We next examined the effect of NMN on uterine receptivity. Aged mice treated with NMN exhibited a higher number of implantation sites (Figure 4I), increased relative uterine weight (Figure 4J), and more uniform spacing of implantations (Figure 4K,L) compared to vehicle‐treated aged mice. Moreover, NMN rescued the expression of ERα in epithelial cells of aged mice (Figure 4M,N). These findings suggest that restoration of uterine NAD^+^ levels improves both endometrial aging and uterine receptivity in aged mice.

To further investigate the transcriptional dynamics underlying the amelioration of endometrial aging by NMN supplementation, we conducted bulk RNA‐seq on uterine tissues from young, aged, and aged mice treated with NMN. Principal‐components analysis (PCA) revealed distinct transcriptomic profiles between the uteri of young and aged mice, whereas the transcriptomic signature of NMN‐treated aged mice was significantly more similar to that of young mice (Figure 4O). Notably, pathways related to ERα downstream signaling, SASP, and inflammatory response were reversed following NMN administration (Figure 4P), supporting the notion that exogenous NMN supplementation reverses endometrial aging in aged mice.

### 2.5 Myeloid‐Specific *Cd38* Ablation Reverses Endometrial Senescent Phenotypes and Restores Receptivity

**FIGURE 5:**
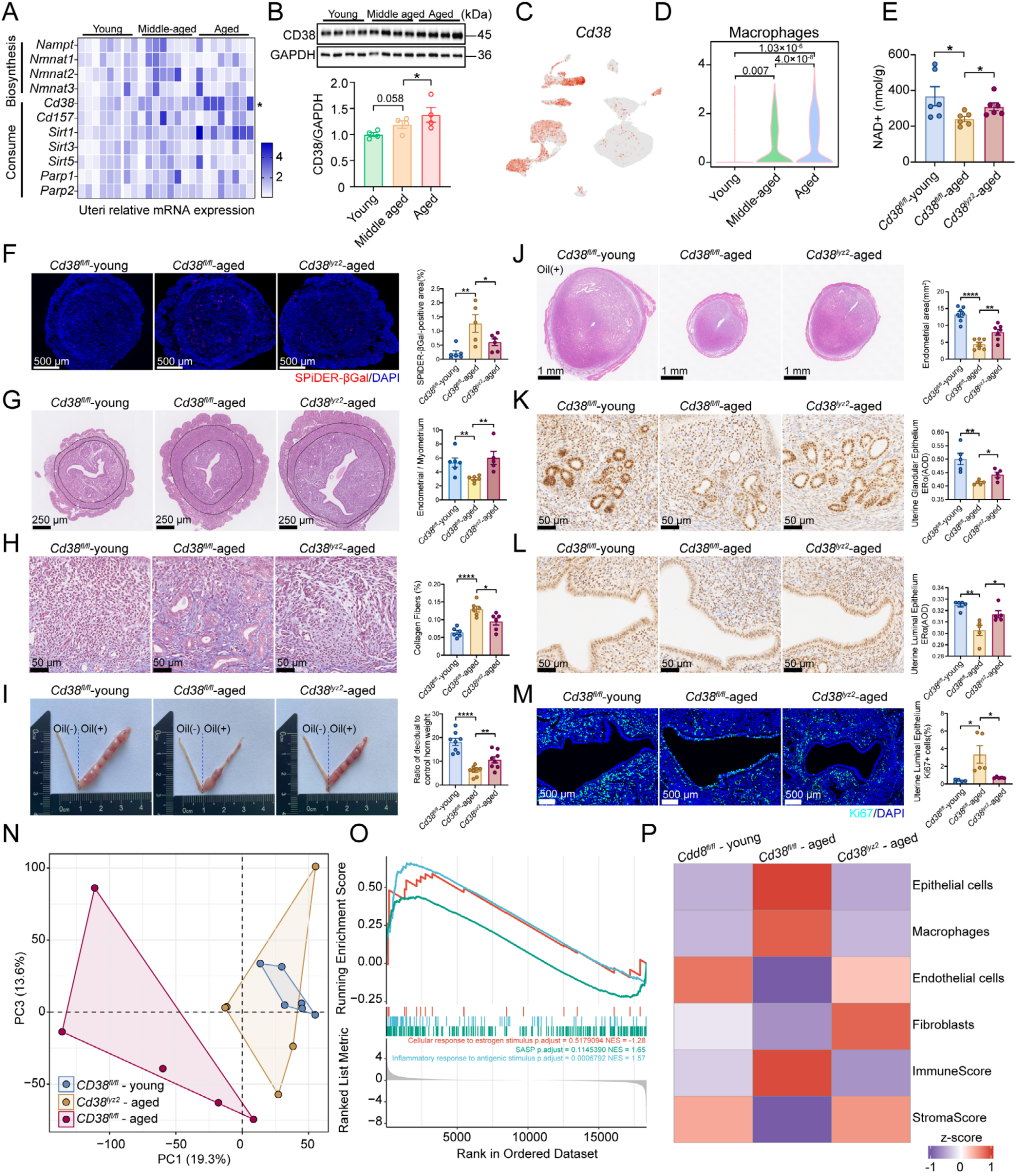
Macrophage‐specific CD38 ablation reverses endometrial senescent phenotypes and restores receptivity pathways. (A) Relative mRNA levels of enzymes involved in NAD^+^‐biosynthesis and consume in the uteri of 3‐, 8‐, and 12‐month‐old mice at the peri‐implantation stage (*n* = 8). (B) Western blot analysis of CD38 in the uteri of 3‐, 8‐, and 12‐month‐old mice. (C, D) UMAP visualization showing Cd38 expression across major cell types in the peri‐implantation uteri of 3‐, 8‐, and 12‐month‐old mice, and violin plot comparing *Cd38* expression in macrophages. (E) NAD^+^ levels in uterine tissues from young WT, aged WT, and age‐matched *Cd38* ^*lyz2*^ mice (*n* = 6). (F) Immunofluorescence staining of SPiDER‐βGal in the endometrium (left) and the quantitative data for the percentage of SPiDER‐βGal‐positive areas (right) in young WT, aged WT, and age‐matched *Cd38* ^*lyz2*^ mice (*n* = 5–6). (G) H&E staining of uteri from young WT, aged WT, and age‐matched *Cd38* ^*lyz2*^ mice. Representative images are shown on the left and quantitative data for the ratio of endometrium‐to‐myometrium are shown on the right (*n* = 5–6). (H) Masson's trichrome staining of uterine tissues from young WT, aged WT, and age‐matched *Cd38* ^*lyz2*^ mice. Representative images are shown on the left and quantitative data of the fibrosis‐positive areas are shown on the right (*n* = 6). (I) Uterine decidua from young WT, aged WT, and age‐matched *Cd38* ^*lyz2*^ mice. Representative images are shown on the left and quantitative data of the endometrial area are shown on the right (*n* = 8). (J) H&E staining of uterine decidua in young WT, aged WT, and age‐matched *Cd38* ^*lyz2*^ mice. Representative images are shown on the left and quantitative data of the endometrium area are shown on the right (*n* = 6). (K, L) IHC staining of ERα in glandular (K) and luminal (L) epithelial cells of the endometrium from young WT, aged WT, and age‐matched *Cd38* ^*lyz2*^ mice. Representative images are shown on the left and quantitative data for the average optical density (AOD) are shown on the right (*n* = 5). (M) Immunofluorescence staining of Ki67 in luminal epithelial cells from young WT, aged WT, and age‐matched *Cd38* ^*lyz2*^ mice. Representative images are shown on the left and quantitative data for the percentage of Ki67‐positive cells are shown on the right (*n* = 5). (N, O) PCA of RNA‐seq data and GSEA analysis of the SASPs and inflammatory response in aged WT and age‐matched *Cd38* ^*lyz2*^ mice. (P) Deconvolution of cell types based on scRNA‐seq reference and cell proportion heatmap from bulk RNA‐seq deconvolution. Data are presented as mean ± SEM. In panels A and B, comparisons among three or more groups were analyzed using one‐way analysis of variance (ANOVA) followed by Tukey's post hoc test. In panels E–M, statistical analysis was performed using an unpaired Student's *t*‐test. Statistical significance was set at *p* < 0.05, and the significance of differences is expressed as: *p* < 0.05 (*), *p* < 0.01 (**), *p* < 0.001 (***), and *p* < 0.0001 (****). Each symbol represents one biological replicate.

To further elucidate the influence of uterine NAD^+^ metabolism, we examined age‐related expression changes of 11 enzymes involved in the NAD^+^ metabolic network (Migaud et al. 2024). Among these enzymes, *Cd38* (a key NAD^+^‐consuming enzyme) exhibited the most pronounced age‐related expression changes (Figure 5A,B). The scRNA‐seq dataset analysis confirmed that the elevated *Cd38* expression was predominantly detected in macrophages of aged mice (Figure 5C,D). These findings strongly suggest that myeloid‐derived *Cd38* may play a pivotal role in the age‐related decline of uterine NAD^+^ levels in mice.

To investigate the impact of myeloid‐derived *Cd38* on endometrial aging, we specifically ablated the *Cd38* gene in mouse macrophages by crossing *Cd38* ^*loxP/loxP*^ mice with *Lyz2*‐Cre transgenic mice. Notably, in myeloid‐specific *Cd38* knockout mice (*Cd38* ^*lyz2*^), NAD^+^ levels in their uteri were significantly higher than those in age‐matched wild‐type (WT) controls (Figure 5E), providing evidence that myeloid‐expressed CD38 is essential for the age‐related reduction of uterine NAD^+^ levels. Consistent with our prior findings of aging‐induced endometrial defects (Figure 1B–D), depletion of *Cd38* in myeloid alleviated these abnormalities. Knockout of *Cd38* in myeloid resulted in decreased SPiDER‐βGal activity (Figure 5F), indicating a potential role of macrophage *Cd38* in regulating cellular senescence. Consistent with this, aged *Cd38* ^*lyz2*^ mice exhibited restored endometrial thickness (Figure 5G), reduced fibrotic deposition (Figure 5H), and enhanced endometrial proliferative capacity compared to age‐matched WT mice (Figure 5I,J). Furthermore, we found elevated ERα levels and ameliorated abnormal proliferation in epithelial cells of myeloid‐specific *Cd38* knockout mice (Figure 5K–M). These results indicate that loss of *Cd38* in myeloid can ameliorate endometrial aging and alleviate aging‐induced impairment of uterine receptivity.

To further investigate the transcriptional alterations in the uterus resulting from *Cd38* depletion in myeloid, we used the bulk RNA‐seq approach. We observed the transcriptomic profiles of *Cd38* ^*lyz2*^ mice are more similar to those of young mice (Figure 5N), and pathways related to ERα downstream signaling, SASP, and inflammatory responses were reversed following myeloid *Cd38* deletion (Figure 5O). These results further support the role of myeloid *Cd38* deletion in alleviating endometrial aging. Deconvolution of bulk RNA‐seq using scRNA‐seq‐derived cell type markers revealed increased myeloid infiltration and decreased stromal cells in the uteri of aged mice (Figure 5P), and these age‐related changes were attenuated in age‐matched mice with *Cd38* depletion in myeloid. Collectively, these results indicate that restoring uterine NAD^+^ levels via myeloid‐specific depletion of *Cd38* attenuates SASP and inflammatory pathways, and reduces immune cell infiltration in aged mice.

## 3 Discussion

**FIGURE 6:**
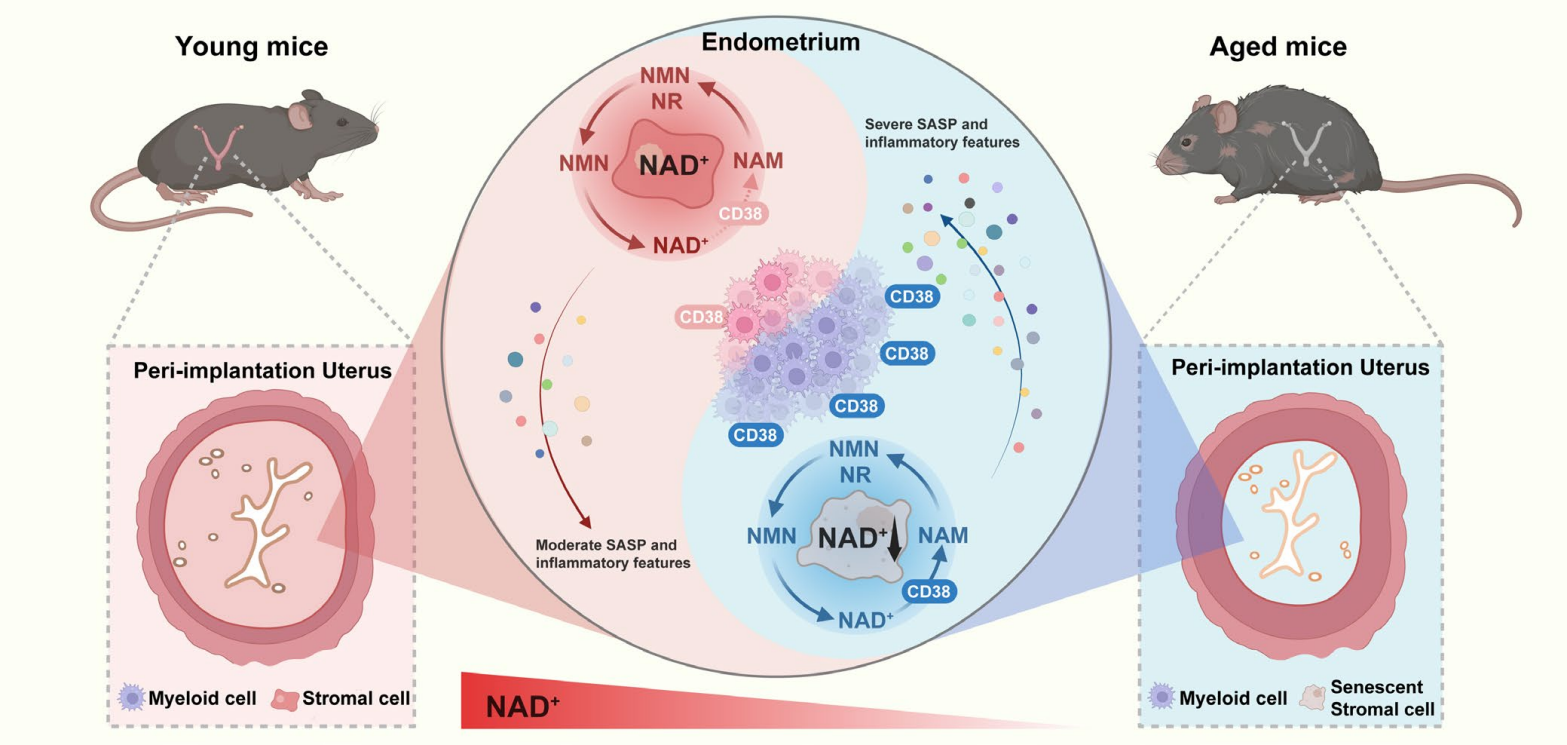
Schematic summary showing that macrophage‐derived CD38 mediates age‐related endometrial aging via NAD^+^ depletion. An increased population of CD38^+^ macrophages, accompanied by a marked decline in stromal cell NAD^+^ levels, serves as key hallmarks of endometrial aging in aged mice. Pharmacological and genetic interventions revealed that macrophage‐derived CD38 is a principal driver of uterine NAD^+^ depletion, thereby accelerating stromal cell senescence and impairing uterine receptivity.

The endometrium serves as the site of embryo implantation (Shibata et al. 2024); therefore, elucidating the mechanisms underlying endometrial aging constitutes a critical biomedical priority for the development of novel therapeutic strategies to address age‐related implantation failure. In this study, we conducted an integrated analysis of scRNA‐seq and metabolomic profiling of mouse uteri across different age groups during the peri‐implantation stage. We identified that an increased population of CD38^+^ macrophages, accompanied by a concurrent decline in NAD^+^ levels, acts as a pivotal signature of endometrial aging in aged mice (Figure 6). Both pharmacological and genetic interventions revealed that myeloid‐expressed CD38 functions as a central driver of uterine NAD^+^ depletion, thereby accelerating endometrial senescence. These findings establish CD38 as a physiological integrator linking NAD^+^ metabolism to uterine function and highlight its potential as a therapeutic target for counteracting endometrial aging.

Female fertility declines sharply with advancing reproductive age, and implantation failure in early pregnancy remains a leading cause of pregnancy loss (Woods et al. 2017; Ruiz‐Alonso et al. 2021). Consistent with previous reports in humans (Rawlings et al. 2021; Loid et al. 2024), we observed an age‐related accumulation of senescent cells in the mouse endometrial epithelium, accompanied by a reduction in endometrial thickness (changes known to impair endometrial receptivity). These alterations were paralleled by a marked depletion of uterine NAD^+^, a metabolic hallmark closely associated with cellular senescence and mitochondrial dysfunction. Notably, our results demonstrate that inflammatory signaling, senescence markers, and NAD^+^ depletion were predominantly localized to stromal cells in aged mice, underscoring their crucial role in maintaining uterine structure and function during decidualization. Interestingly, NAD^+^ levels in epithelial cells, the site of embryo attachment, remained stable with age, in contrast to the overall endometrial tissue. By comparison, stromal cells, the predominant cell type in the uterus, exhibited age‐associated NAD^+^ decline, indicating that their NAD^+^ metabolism is particularly susceptible to aging. The high metabolic activity of epithelial cells during the implantation window (Garcia‐Alonso et al. 2021) further suggests that cells with proliferative and differentiative capacity may be more resilient to age‐related NAD^+^ loss.

Restoration of uterine NAD^+^ levels in aged mice improved both histological and functional indices of endometrial aging, thereby enhancing receptivity. These findings underscore that age‐related and pregnancy‐associated remodeling of uterine NAD^+^ metabolism is central to successful decidualization and implantation in aged females. Although NMN supplementation restored uterine NAD^+^ levels to the range observed in young mice, the recovery of decidualization remained partial. This observation suggests that the decline in NAD^+^ level is a contributing factor to endometrial aging, rather than the sole determinant. This process likely arises from a combination of metabolic disorders, epigenetic modifications, and structural impairments associated with advancing age (Wang et al. 2025). It is noteworthy that reduced NAD^+^ levels may not be the exclusive determinant of age‐related uterine functional deterioration. Decreased sensitivity to estrogen and progesterone has also been documented to compromise endometrial receptivity (Wang et al. 2025). Consequently, investigating the interplay between estrogen and progesterone signaling pathways and NAD^+^ metabolism during the implantation window would be of considerable interest.

During implantation, the maternal‐fetal interface requires a tightly regulated state of immune tolerance, primarily mediated by leukocytes such as macrophages, NK cells, and T cells (Samstein et al. 2012; Zhou et al. 2022; Chen et al. 2024; Hosking et al. 2024; Li et al. 2024). Our data reveal that aging is associated with increased infiltration of macrophages and upregulation of the NAD^+^ glycohydrolase, CD38 in the uterus. Senescent stromal cells are known to secrete SASP factors, including IL‐6, CCL2, and CXCL10, which may recruit and activate macrophages (Huang et al. 2022; Taylor et al. 2024; Wang et al. 2024). CD38 is a well‐characterized regulator of tissue NAD^+^ homeostasis (Malavasi et al. 2008; Wang et al. 2022; Zeidler et al. 2022). In the ovary, pro‐inflammatory cytokines promote CD38 expression, forming a feed‐forward loop that exacerbates NAD^+^ depletion, thereby accelerating ovarian aging (Yang, Chen, et al. 2024; Yang, Liu, et al. 2024; Tarragó et al. 2018; Chini et al. 2020; Covarrubias et al. 2020). These findings suggest that CD38‐mediated NAD^+^ depletion may contribute to the female reproductive system being one of the first organ systems to undergo age‐related deterioration. In this study, we found that elevated levels of CD38 were predominantly expressed in macrophages of aged mice. Consistent with this observation, selective depletion of *Cd38* in myeloid effectively restored uterine NAD^+^ levels, improved histological parameters of endometrial aging, and enhanced uterine receptivity in aged mice. Thus, we hypothesize that CD38‐mediated NAD^+^ loss in stromal cells leads to impaired mitochondrial biogenesis and increased ROS production. These changes could reinforce cellular senescence and hinder decidualization. Our morphological and scRNA‐seq analyses confirmed the profound senescent features of endometrial stromal cells in aged mice. In addition, NAD^+^ depletion may dysregulate PARP activity, further compromising DNA repair capacity during the implantation window (Cohen 2020; Pollard et al. 2022). Taken together, our data support the notion that increased CD38 expression in myeloid precipitates a localized NAD^+^ crisis in the uterine stroma, thereby impairing structural remodeling and endometrial receptivity.

Elevated activity of NADases CD38 contributes to mitochondrial dysfunction (Covarrubias et al. 2021; Chini et al. 2024), which is closely associated with impaired uterine receptivity. Blocking the ecto‐enzymatic activity of CD38 can increase NAD^+^ levels through a nicotinamide mononucleotide (NMN)‐dependent process (Chini et al. 2024). Thus, the effects of CD38 on mitochondrial function may be mediated, at least in part, by modulating the availability of NAD^+^ as a substrate for mitochondrial enzymes. Given that CD38 regulates both global cellular and mitochondrial NAD^+^ levels, its effects may also be mediated by interfering with multiple other NAD^+^‐dependent cellular processes, including redox reactions, signal transduction, and epigenetic regulation.

In conclusion, we identify NAD^+^ deficiency and elevated CD38 expression as defining signatures of endometrial senescence in aged mice. Our findings demonstrate that myeloid‐derived CD38 promotes NAD^+^ depletion, thereby impairing uterine function during the peri‐implantation period. These insights offer a valuable therapeutic avenue for extending reproductive lifespan and counteracting age‐related decline in female fertility.

## 4 Materials and Methods

### 4.1 Animal Feeding and Treatments

All experimental procedures were conducted in accordance with the ethical guidelines approved by the Animal Ethics and Welfare Committee of Sichuan Agricultural University (No. 20240154). 3, 8, and 12 months C57BL/6J female mice were obtained from Newlong Youshu Life Technology (Hangzhou) Co. Ltd. (Hangzhou, China). We generated myeloid‐specific *Cd38* knockout mice (*Cd38* ^*lyz2*^) by breeding *Cd38* ^*loxP/loxP*^ mice (Cyagen, CKOAIP221017RM1) with *Lyz2*‐Cre mice (Cyagen, C001003).

Female mice were caged with fertile male mice at a ratio of 1:1 at 20:00. Female mice with vaginal plugs observed the next morning were marked as embryonic Day 1 (E1). All mice were housed under conditions of a constant temperature of 20°C–22°C, a constant humidity of 45%–65%, and a 12‐h dark/light cycle, with free access to food and water.

Mice in the NMN group received daily intraperitoneal injections of 200 mg/kg BW for 2 weeks before mating. The control group was injected with PBS buffer for the same duration. After collection of uterine samples, a portion was fixed in 4% paraformaldehyde, and the remainder was stored at −80°C.

### 4.2 Artificial Decidualization

To assess endometrial stromal cell proliferation and differentiation independent of implantation, ovariectomized mice were hormonally primed to mimic pregnancy. After a 2‐week recovery, mice received daily subcutaneous injections of estradiol (E_2_, 100 ng; Selleck, S1709) for 3 days, followed by 2 days of withdrawal. They were then treated with progesterone (P_4_, 1 mg; Selleck, S1705) plus E_2_ (10 ng) for 3 days. Six hours after the last injection, 35 μL of corn oil was infused into the right uterine horn. Hormone treatment (P_4_ plus E_2_) was continued for 4 additional days, after which mice were euthanized and the wet weights of stimulated and contralateral horns were recorded.

### 4.3 Embryo Implantation Sites and Spacing

To assess embryo implantation sites, mice were injected via the tail vein with 30 μL of 2% Chicago Sky Blue 6B (Yuanye, 2610‐05‐1) on the morning of embryonic Day 6 and euthanized 30 min later. Uteri were collected and imaged, and the number of implantation sites per uterus was recorded. Blastocyst spacing (distance between adjacent implantation sites) was measured using ImageJ. The standard deviation (SD) of blastocyst spacing within each uterus was calculated, with higher SD values indicating uneven distribution.

### 4.4 Mice Primary Cells Isolation and Treatment

Uterine tissues were rinsed in PBS, incised longitudinally, and digested with trypsin (Gibco, 25200072) to isolate epithelial cells. Residual tissues were digested with 2 mg/mL collagenase II (Worthington Biochemical Corporation, LS004176), filtered, and centrifuged to obtain stromal cells, which were cultured in phenol red‐free DMEM/F‐12 (Gibco, 11039021) with 10% FBS (Gibco, 10091‐148). Non‐adherent cells were removed after 2 h, and medium was refreshed every 48 h.

For in vitro decidualization, stromal cells at ~80% confluence were cultured in induction medium containing 10 nM E_2_ (Selleck, S1709) and 1 μM P_4_ (Selleck, S1705) for 5 days. Stromal cells undergoing decidualization were treated with NR (100 μM) or NMN (100 μM) throughout the 5‐day induction period, and the inducing medium was replaced every 48 h.

### 4.5 Cell Senescence Staining

Cell senescence staining was performed using the Cell Senescence β‐Galactosidase Staining Kit (Beyotime, C0602) following the manufacturer's instructions. Briefly, after aspirating the culture medium, cells were washed once with PBS, fixed with 1 mL of fixation solution at room temperature for 15 min, and the fixative was aspirated followed by three PBS washes. The staining working solution was added (1 mL/well), and the plate was sealed and incubated at 37°C overnight, then observed under microscopy (Nikon 80i).

### 4.6 Tissue Histology and Immunostaining

Uterine tissues were fixed in 4% paraformaldehyde, paraffin‐embedded, and sectioned for H&E staining, with tissue morphology quantified using ImageJ. For immunohistochemistry, sections were dewaxed, antigen‐retrieved in citrate buffer, blocked with 3% H_2_O_2_ and serum, incubated with ERα antibody (Abcam, AB32063) overnight at 4°C, followed by secondary antibody at 37°C for 45 min, and visualized with diaminobenzidine (DAB) and hematoxylin. For immunofluorescence, sections were incubated with Ki67 antibody (CST, 9129S) overnight and counterstained with DAPI (Solarbio, C0060). Bright‐field and fluorescence images were acquired using NanoZoomer S360 and Pannoramic SCAN II, respectively.

### 4.7 Masson Staining

Masson's trichrome staining was performed using a commercial kit (Solarbio, G1340) following the manufacturer's protocol. Briefly, sections were stained with hematoxylin, differentiated with hydrochloric acid alcohol, and blued with lithium carbonate. After staining with ponceau‐acid fuchsin, the sections were treated with phosphomolybdic acid followed by counterstaining with aniline blue. Finally, the sections were treated with glacial acetic acid, dehydrated, cleared, and mounted.

### 4.8 SPiDER‐βGal and Immunofluorescence Co‐Staining

Uterine senescence staining was performed using the SPiDER‐βGal kit (Dojindo Laboratories, Japan, SG02). Freshly isolated uterine tissues were rapidly frozen using dry ice, embedded in OCT, and then processed for frozen sectioning. Frozen sections were treated with antigen retrieval solution for frozen sections at room temperature for 10 min, permeabilized with 0.1% Triton X‐100 (BioFroxx, 1139ML100) for 15 min, and washed three times with TBST. Subsequently, the sections were incubated with SPiDER‐βGal (11.4 ug/mL) at 37°C for 1 h. After removing TBST, the sections were incubated with DAPI (Solarbio, C0060) in the dark for 5 min, rinsed with TBST, mounted, and examined under a microscope (Olympus, BX53).

### 4.9 Determination of NAD ^+^ Content

We extracted NAD^+^ from uterine tissues and cells using the Coenzyme I NAD(H) Content Detection Kit (Solarbio, bc0315) per the manufacturer's instructions. For tissues, acidic extraction solution was added for homogenization; the supernatant was mixed with an equal volume of alkaline extraction solution, detected via standard absorbance, and NAD^+^ content calculated by uterine weight. For uterine epithelial and stromal cells, acidic solution was added by cell number; after ultrasonic disruption, equal alkaline solution was added, detected with standard absorbance, and NAD^+^ content calculated by cell number.

### 4.10 Metabolite Profiling

Polar metabolites were extracted from ~50 mg of tissue using 500 μL ice‐cold methanol:H_2_O (4:1, v/v) containing 0.224 mM phenylhydrazine. Samples were minced and homogenized using a bead beater. Homogenates were centrifuged at 1500 rpm for 30 min at 4 C, then incubated at −20°C for 1 h for α‐keto acid derivatization. A second centrifugation at 12,000 rpm for 10 min at 4 C followed. The extraction was repeated with 500 μL ice‐cold methanol + phenylhydrazine, and pooled extracts were dried in a SpeedVac. Dried residues were reconstituted in 5% acetonitrile for LC–MS analysis using an Agilent 1290 II UPLC and Sciex 5600+ QTOF‐MS. Metabolites were separated using Waters ACQUITY HSS‐T3 (3 × 100 mm, 1.8 μm) or BEH Amide (2.1 × 100 mm, 1.7 μm) columns. Isotopically labeled internal standards (Cambridge Isotope Laboratories) were used for quantification. Endogenous metabolite peaks were normalized to corresponding internal standards; in the absence of structural analogues, normalization was based on minimal coefficient of variation.

### 4.11 Western Blotting

We added cold RIPA buffer containing a protease inhibitor (Sigma‐Aldrich) to the tissues and homogenized the tissues using a FastPrep‐24 5G (MP Biomedicals). Subsequently, the lysates were centrifuged at 12,000 rpm at 4°C for 30 min, and the proteins were then separated on an SDS‐PAGE gel. We electrotransferred the proteins onto PVDF membranes and probed them with antibodies against GAPDH (Proteintech, 10494‐1‐AP) and CD38 (CST, 68336). The protein level of each sample was normalized to that of GAPDH.

### 4.12 Quantitative PCR (q‐PCR) Analysis

We extracted total RNA using TRIzol reagent (Thermo Fisher Scientific). We synthesized cDNA from 1 μg of RNA and performed q‐PCR in a 10 μL reaction volume using SYBR Green. The relative expression levels were determined by the ΔCT method and normalized to *β*‐*Actin*. All PCR primers are listed in the Table S1.

### 4.13 Single‐Cell RNA Sequencing

#### 4.13.1 Library Preparation

Minced tissues were digested for 30 min at 37°C in the enzymatic solution. The single‐cell suspensions were filtered through a 70‐μm and 30‐μm cell strainer (Miltenyi Biotech, PN: 130‐098‐458 and 130‐098‐462), and then treated with the Red Blood Cell Lysis Solution (Miltenyi Biotech, PN: 130‐094‐183). The viability of cells was determined by using Countstar Rigel (Alit Biotech), and dead cell removal was carried out depending on the viability using the Dead Cell Removal Kit (Miltenyi Biotech, PN: 130‐090‐101). Finally, the cells were re‐suspended in 1× PBS (Invitrogen) supplemented with 0.04% BSA at a final concentration of 700–1200 cells/μL, and were then processed with the MobiCube High Throughput Single Cell 3′ RNA‐Seq Kit v2.1 (MobiDrop, PN: S050200301) as per the manufacturer's instructions at Novogene Bioinformatics Technology Co. Ltd. (Tianjin, China).

#### 4.13.2 Preprocessing, Clustering, and Cell Type Annotation

Raw sequencing data were aligned to the GRCm39 reference genome using MobiVision (v3.2) to generate count matrices. A total of 76,233 cells across nine samples were profiled. Ambient RNA contamination was removed using SoupX (v1.6.2) (Young and Behjati 2020), and doublets were filtered out with scDblFinder (v1.16.0) (Germain et al. 2021).

Cells were retained if they met the following quality thresholds: (i) > 350 expressed genes; (ii) > 1000 UMIs; (iii) mitochondrial gene fraction below the median plus five median absolute deviations (MADs). Genes expressed in fewer than 20 cells were also excluded. After filtering, 63,979 high‐quality cells remained.

Data integration across the nine libraries was performed using Seurat (v5.1.0) (Hao et al. 2024). Counts were normalized via NormalizaData and scaled via the ScaleData function, regressing out mitochondrial content as well as cell cycle effects. Batch effects were corrected using harmony based on the top 2000 highly variable genes, implemented via Seurat's IntegrateLayers function.

Dimensionality reduction was performed using harmony, and the top 30 dims were used to construct a shared nearest neighbor (SNN) graph via the FindNeighbors function. Clustering was performed using the FindClusters function, and visualization was achieved via UMAP (RunUMAP function). Cell clusters were annotated using known lineage‐specific markers from previous uterus studies. Differential gene expression for each cluster was calculated using the Wilcoxon rank‐sum test with thresholds of avg_logFC > 0.25, adjusted *p* < 0.05, and min.pct > 0.20.

Immune cells were sub‐clustered at resolution 0.2, stromal cells at 0.2 using integrated analysis methods. Cells within the immune‐cell subclusters that exhibited high expression of stromal cell marker genes were classified as contaminating cells and excluded from all subsequent analyses. In total, 63,083 cells were retained for downstream analysis.

#### 4.13.3 Analysis of Cell Type Composition and Abundance

Differential abundance analysis was conducted using miloR (v1.10.0) (Dann et al. 2022), a k‐nearest neighbor graph‐based framework. To ensure balanced comparisons between any two groups, samples were down‐sampled to equal cell numbers. A SingleCellExperiment object was created from the count matrix. Neighborhood graphs were constructed using buildGraph (*k* = 30, *d* = 50) and makeNhoods (prop = 0.2, *k* = 30, *d* = 50, refinement_scheme = ’graph’).

Differential abundance was tested using testNhoods, with significant neighborhoods (spatial FDR < 0.1) visualized via plotDAbeeswarm function. Changes in cell type composition were quantified by computing the local true sign rate (LTSR) (Young et al. 2021), which estimates the probability that the direction of log_2_ fold change is correct given its mean and variance. A threshold of LTSR > 0.9 was used to define significantly altered cell types.

#### 4.13.4 Signature Score Definition

To evaluate transcriptional changes associated with uterus aging, we computed a SASP score using a predefined SASP gene set. Gene set activity scores were calculated with the AddModuleScore function of Seurat. Additional scores for inflammatory response, DNA repair, and estrogen receptor signaling pathway were derived using curated gene sets from the Gene Ontology and KEGG databases.

#### 4.13.5 Aging Sensibility Analysis

The prioritization of cell types in the response to uterus aging was calculated and named as Augur score using calculate_auc function from Augur package (v1.0.3) (Skinnider et al. 2021) by inputting the genes‐by‐cells scRNA‐seq matrix and a data frame containing cell type and aged group columns.

#### 4.13.6 Transcriptional Noise Analysis

We employed a method to analyze the impact of aging on various cell types through transcriptional noise, building upon previous studies (Angelidis et al. 2019). Transcriptional noise was quantified for each cell type by analyzing a minimum of 10 cells from both aged stages. To standardize for differences in total UMI counts, all cells were down sampled to ensure equal library sizes. Additionally, to account for variations in cell‐type frequency, scRNA‐seq data were down sampled to 300 cells per cell type in each age group; cell types with fewer than 300 cells included all available cells. Next, genes were categorized into 10 blocks based on their average expression levels, and genes with the lowest coefficient of variation (CV) in each block (the lowest 10% CV) were selected. The Euclidean distance between each cell was then calculated based on these genes, serving as a measure of transcriptional heterogeneity within each cell type.

#### 4.13.7 Senescent Cell Identification

To identify senescent cells, we devised a unified senescence score (USS) algorithm building on previous studies (Li et al. 2025). USS integrates four established senescence gene databases (SenMayo [SM], CellAge [CA], GenAge [GA], and the Senescence Eigengene approach [SE]) and two additional gene sets: the senescence‐associated secretory phenotype (SASP) and an inflammatory response (IR). All cells were first divided based on cell types. Within the same cell type, ss‐GSVA score was calculated for each cell with GSVA package (v1.50.5) using the six different gene sets. ss‐GSVA scores SM, CA, GA, SE, SASP, and IR into two halves by the median value, and senescent cells were defined as those possessing ss‐GSVA scores in the upper half level.

### 4.14 Library Preparation for Bulk RNA‐Seq Analysis

Total RNA was extracted using TRIzol reagent (Invitrogen) according to the manufacturer's protocol. RNA concentration and purity were assessed with a NanoDrop ND‐1000 spectrophotometer, while integrity was verified using a Bioanalyzer 2100 (Agilent), ensuring RNA integrity numbers (RIN) exceeded 7.0. Electrophoresis on a denaturing agarose gel was used to further confirm integrity. From each sample, 1 μg of total RNA was subjected to two rounds of poly(A) selection using Dynabeads Oligo (dT)25‐61,005 (Thermo Fisher). The enriched poly(A) RNA was fragmented with the Magnesium RNA Fragmentation Module (NEB) at 94 C for 5–7 min.

First‐strand cDNA synthesis was performed using SuperScript II Reverse Transcriptase (Invitrogen), followed by second‐strand synthesis using *E. coli* DNA polymerase I, RNase H, and dUTP (NEB and Thermo Fisher). A‐tailing was performed to add an adenine overhang at the 3′ ends of blunt‐ended fragments. Indexed adapters with complementary thymine overhangs were ligated to the A‐tailed fragments. Both single‐ and dual‐index adapters were used. Adapter‐ligated fragments were size‐selected using AMPure XP beads. Subsequently, second‐strand U‐labeled DNA was degraded using heat‐labile Uracil‐DNA Glycosylase (NEB). Libraries were PCR amplified under the following thermal cycling conditions: initial denaturation at 95 C for 3 min; 8 cycles of 98°C for 15 s, 60 C for 15 s, and 72 C for 30 s; followed by a final extension at 72 C for 5 min. The final cDNA libraries had an average insert size of 300 ± 50 bp. Paired‐end sequencing (PE150) was conducted on an Illumina NovaSeq 6000 (LC‐Bio Technology Co. Ltd.) following the standard protocol.

#### 4.14.1 Quality Control, Alignment, and Differential Gene Expression Analysis

Transcript quantification of high‐quality data was carried out using kallisto (v0.48.0) against the GRCm39 reference genome (GCF_000001635.27) and Ensembl v110 annotations (Bray et al. 2016). Transcript‐level abundance estimates were aggregated to gene‐level TPMs using the tximport R package (v1.30.0). Protein‐coding genes with TPM > 0.1 in at least 50% of replicates per condition were retained. Differential gene expression analysis was performed using DESeq2 (v1.42.1) (Love et al. 2014) with the Wald test. Adjusted *p*‐values (*p*adj) were calculated using Independent Hypothesis Weighting (IHW) (Ignatiadis et al. 2016), and log_2_ fold changes were stabilized using the apeglm algorithm (Zhu et al. 2019).

#### 4.14.2 Principal Component Analysis

Principal component analysis (PCA) was conducted using FactoMineR (v2.11) and factoextra (v‐1.0.7), visualizing sample distributions in PC1 versus PC2 space.

#### 4.14.3 Gene Set Enrichment Analysis (GSEA)

Gene set enrichment analysis (GSEA) was performed using the clusterProfiler (v‐4.10.1) R package on a pre‐ranked gene list (Wu et al. 2021). Genes were ranked by the DE statistic (DESeq2 stat).

#### 4.14.4 Deconvolution of Bulk RNA‐Seq

Bulk RNA‐seq deconvolution was carried out using CIBERSORTx (Steen et al. 2020), employing cell‐type‐specific expression profiles from our scRNA‐seq dataset as the reference signature matrix.

#### 4.14.5 Gene Ontology (GO) Enrichment Analysis

Gene Ontology enrichment of differentially expressed genes was performed using Metascape (Zhou et al. 2019).

### 4.15 Statistical Analysis

Perform statistical analysis on GraphPad Prism 6 software. Data are presented as mean ± SEM. Comparisons between two groups were analyzed using unpaired Student's *t*‐test. Comparisons between three or more groups were analyzed using one‐way analysis of variance (ANOVA) followed by Tukey's post hoc testing. The statistical significance is set to *p* < 0.05, and the difference significance is expressed as: *p* < 0.05 (*), *p* < 0.01 (**), *p* < 0.001 (***), *p* < 0.0001 (****).

## Author Contributions

L.H., M.L., and D.W. designed the research. L.H., L.L., D.G., X.J., L.M., L.L., S.T., X.J., C.J., and B.F. performed the research. D.G., L.C., S.X., Y.L., Y.Z., and L.J. analyzed the data. L.H., M.L., and D.W. wrote the paper. All authors reviewed and edited the manuscript.

## Funding

This work was supported by the National Natural Science Foundation of China (32230102 and 32421005 to De Wu, 32225046 and 32494802 to M.L., 32472948 to L.H.), the Science and Technology Projects of Xizang Autonomous Region of China (XZ202501ZY0147 to M.L.), the Agricultural Science and Technology Major Project (to M.L.), and the earmarked fund for China Agriculture Research System (CARS‐35 to L.C.).

## Ethics Statement

All experimental procedures were conducted in accordance with the ethical guidelines approved by the Animal Ethics and Welfare Committee of Sichuan Agricultural University (No. 20240154).

## Conflicts of Interest

The authors declare no conflicts of interest.

## Supporting information


**Figure S1:** Additional scRNA‐seq data.
**Figure S2:** Additional senescence atlas of scRNA‐seq data.
**Figure S3:** Expression profiling of *Cd38* across major cell types in peri‐implantation uteri of mice across stages.
**Table S1:** Primers used for qPCR.

## Data Availability

All high‐throughput sequencing datasets are deposited at the Genome Sequence Archive (GSA) (https://ngdc.cncb.ac.cn/gsa/) of the China National Center for Bioinformation National Genomics Data Center (CNCB‐NGDC) under accession code CRA030134. All additional datasets included in the manuscript will be provided upon request from the lead contact. All the custom codes are available from the corresponding authors upon reasonable request.
