# Supplementary material for: Myeloid‐Derived CD38 Mediates Age‐Related Endometrial Aging Through NAD + Depletion
Source: Aging Cell. 2025 Dec 28;25(1):e70356. doi: 10.1111/acel.70356 (PMC12745663; doi:10.1111/acel.70356)
Supplement: Supplementary file 1 — Figure S1: Additional scRNA‐seq data. Figure S2: Additional senescence atlas of scRNA‐seq data. Figure S3: Expression profiling of Cd38 across major cell types in peri‐implantation uteri of mice across stages. Table S1: Primers used for qPCR. [file ACEL-25-e70356-s001.docx]

**Supplementary Figure**

**
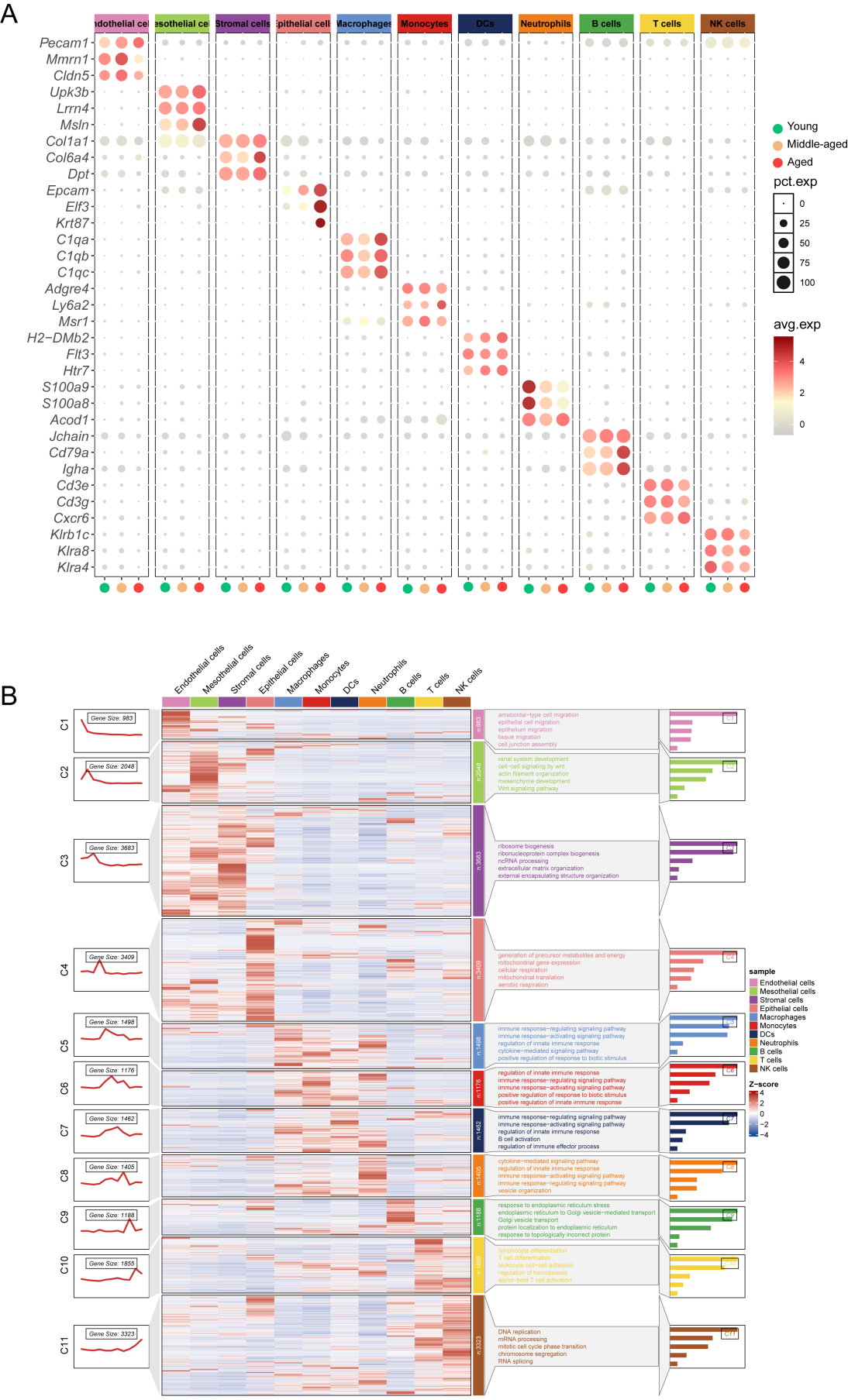
**

**Figure S1. Additional scRNA-seq data.**

1. Dot plot showing the expression of canonical marker genes for each cell type in the scRNA-seq dataset of uteri from 3-, 8-, and 12-month-old mice.

(B) Functional enrichment analysis of cell-type-specific genes. Left: Expression dynamics of the identified marker genes across the three age groups. Left-middle: Marker gene expression across different cell types. Right-middle: Top five significant GO-BP terms associated with highly expressed genes in each cell type. Right: GO-BP terms ranked by -log₁₀(*P*) values.

**
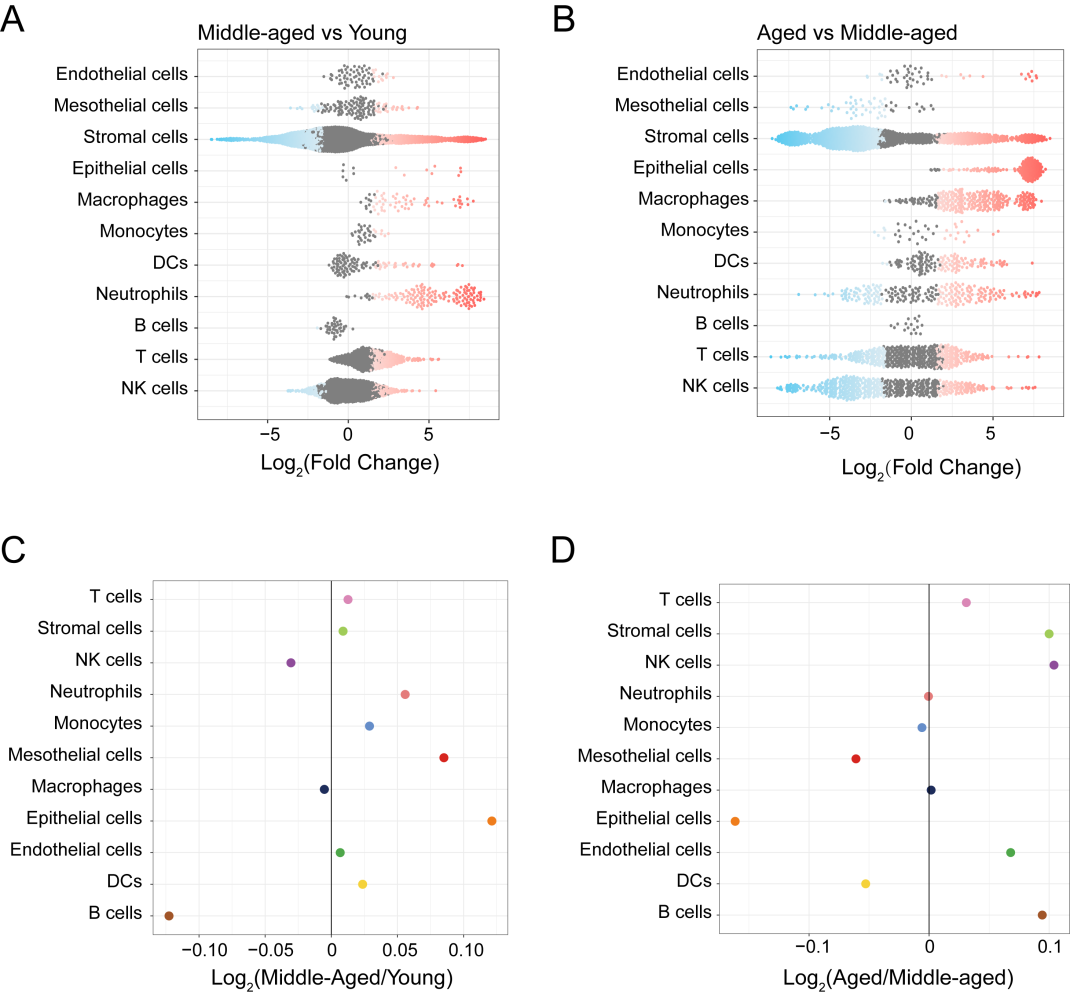
**

**Figure S2. Additional senescence atlas of scRNA-seq data.**

(A, B) Bee-swarm plot of log₂ fold changes in cell-type abundance from Milo analysis. Neighborhoods with a spatial FDR ≤ 10% are colored.

(C, D) Dot plots showing the log_2_ ratio of transcriptional noise.


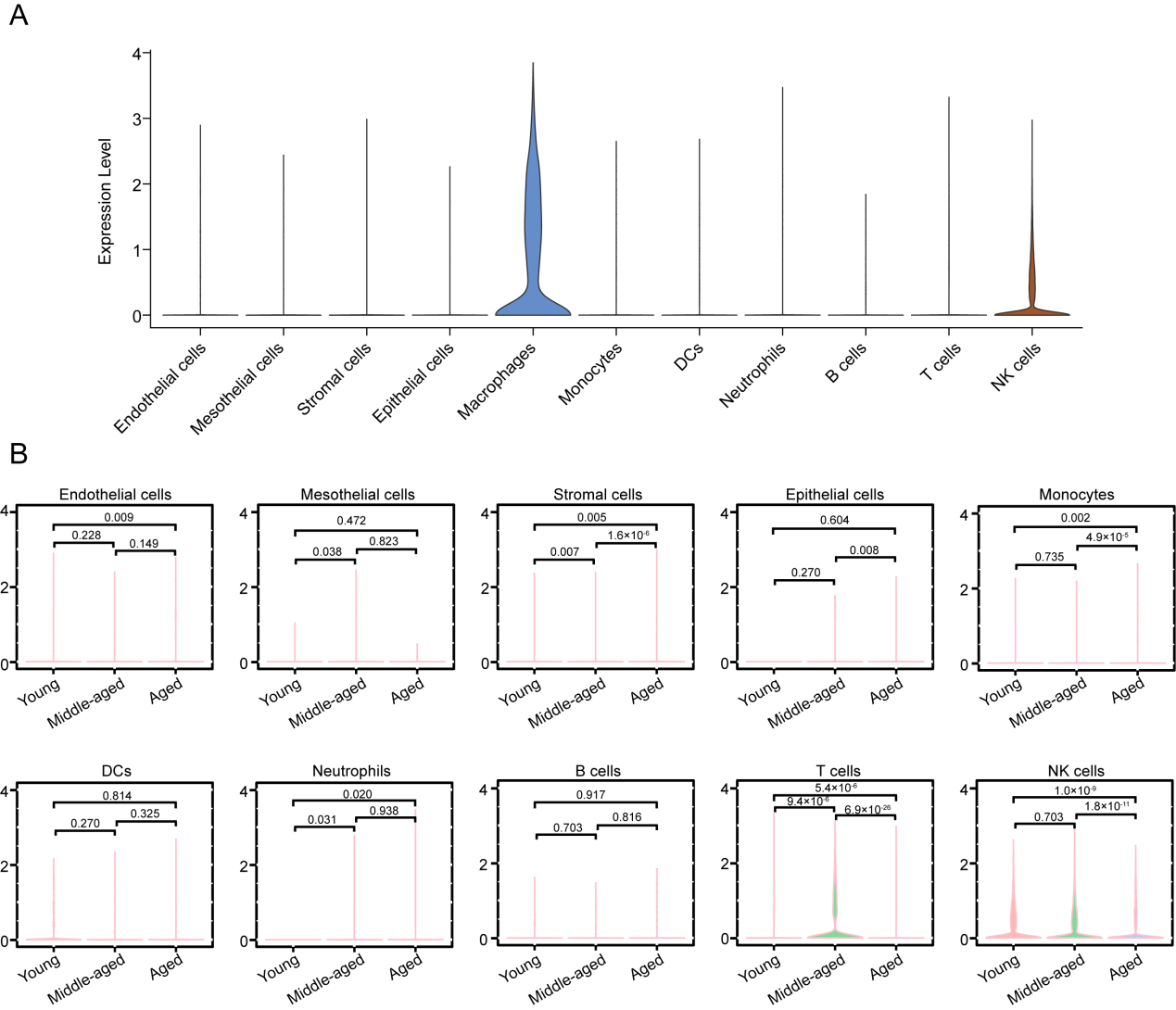


**Figure S3. Expression profiling of *Cd38* across major cell types in peri-implantation uteri of mice across stages.**

(A) Comparison of *Cd38* expression levels among distinct major cell types.

(B) Violin plot showing the comparison of *Cd38* expression profiles across major cell types in the peri-implantation uteri of 3-, 8-, and 12-month-old mice.

Table S1 . Primers used for qPCR

| Genes | Forward(5'-3') | Reverse(5'-3') |
| --- | --- | --- |
| *β-Actin* | GGCTGTATTCCCCTCCATCG | CCAGTTGGTAACAATGCCATGT |
| *Nampt* | CCCGATTGAAGTAAAGGCTGT | TGGTAAGCCAGTAGCACTCTG |
| *Nmnat1* | TGGCTCTTTTAACCCCATCAC | TCTTCTTGTACGCATCACCGA |
| *Nmnat2* | ATGACCGAGACCACAAAGACC | ATCCCGCCAATCACAATAAATCT |
| *Nmnat3* | TCACCCGTCAATGACAGCTAT | CACCCGAATCCAGTCAGATGT |
| *Cd38* | TCTCTAGGAAAGCCCAGATCG | AGAAAAGTGCTTCGTGGTAGG |
| *Cd157* | AGGGACAAGTCACTGTTCTGG | AACTTTGCCATACAGCACGTC |
| *Sirt1* | GCTGACGACTTCGACGACG | TCGGTCAACAGGAGGTTGTCT |
| *Sirt3* | GGCTCTATACACAGAACATCGAC | TAGCTGTTACAAAGGTCCCGT |
| *Sirt5* | CCAGTTGTGTTGTAGACGAAAGC | TTCCGAAAGTCTGCCATATTTGA |
| *Parp1* | GGAGCTGCTCATCTTCAACC | GCAGTGACATCCCCAGTACA |
| *Parp2* | GGAAGGCGAGTGCTAAATGAA | AAGGTCTTCACAGAGTCTCGATTG |
| *Prl8a2* | AGCCAGAAATCACTGCCACTC | TGATCCATGCACCCATAAAA |
| *Prl3c1* | GCCACACGATATGACCGGAA | GGTTTGGCACATCTTGGTGTT |
| *Bmp2* | GTTTGGCCTGAAGCAGAGAGA | GTCGAAGCTCTCCCACTGAC |
| *Cebpb* | GTTTCGGGACTTGATGCAAT | CCCGCAGGAACATCTTTAAGT |
| *Hoxa10* | ATGAGTCAAGGCAGTTCCA | CCAGCGTCTGGTGCTTCGT |
